# The triply periodic minimal surface-based 3D printed engineering scaffold for meniscus function reconstruction

**DOI:** 10.1186/s40824-022-00293-3

**Published:** 2022-09-17

**Authors:** Lan Li, Peng Wang, Jing Jin, Chunmei Xie, Bin Xue, Jiancheng Lai, Liya Zhu, Qing Jiang

**Affiliations:** 1 State Key Laboratory of Pharmaceutical Biotechnology, Division of Sports Medicine and Adult Reconstructive Surgery, Department of Orthopedic Surgery, Branch of National Clinical Research Center for Orthopedics, Drum Tower Hospital Affiliated to Medical School of Nanjing University, No.321 Zhongshan Road, Nanjing, 210000 China; 2 Jiangsu Engineering Research Center for 3D Bioprinting, Nanjing, 210000 China; 3 Hangzhou Lancet Robotics Company Ltd, Hangzhou, 310000 China; 4 National Laboratory of Solid State Microstructures, Department of Physics, Nanjing University, Nanjing, 210093 China; 5 Department of Chemical Engineering, Stanford University, Stanford, CA 94305-6104 USA; 6 School of Electrical and Automation Engineering, Nanjing Normal University, No.1 Wenyuan Road, Nanjing, 210023 China

**Keywords:** Meniscal scaffold, Triply periodic minimal surface, Mechanical properties, Cartilage protection

## Abstract

**Background:**

The meniscus injury is a common disease in the area of sports medicine. The main treatment for this disease is the pain relief, rather than the meniscal function recovery. It may lead to a poor prognosis and accelerate the progression of osteoarthritis. In this study, we designed a meniscal scaffold to achieve the purposes of meniscal function recovery and cartilage protection.

**Methods:**

The meniscal scaffold was designed using the triply periodic minimal surface (TPMS) method. The scaffold was simulated as a three-dimensional (3D) intact knee model using a finite element analysis software to obtain the results of different mechanical tests. The mechanical properties were gained through the universal machine. Finally, an in vivo model was established to evaluate the effects of the TPMS-based meniscal scaffold on the cartilage protection. The radiography and histological examinations were performed to assess the cartilage and bony structures. Different regions of the regenerated meniscus were tested using the universal machine to assess the biomechanical functions.

**Results:**

The TPMS-based meniscal scaffold with a larger volume fraction and a longer functional periodicity demonstrated a better mechanical performance, and the load transmission and stress distribution were closer to the native biomechanical environment. The radiographic images and histological results of the TPMS group exhibited a better performance in terms of cartilage protection than the grid group. The regenerated meniscus in the TPMS group also had similar mechanical properties to the native meniscus.

**Conclusion:**

The TPMS method can affect the mechanical properties by adjusting the volume fraction and functional periodicity. The TPMS-based meniscal scaffold showed appropriate features for meniscal regeneration and cartilage protection.

**Supplementary Information:**

The online version contains supplementary material available at 10.1186/s40824-022-00293-3.

## Background

A common inducement of knee cartilage damage is ineffective treatment after meniscus injury. Meniscus is a pair of crescentic fibrocartilages, which is located between the two load-bearing articular surfaces of femur and tibia. It plays an important role in mechanical shock absorption, load transmission, and auxiliary lubrication in the knee joint [1]. During suffering load, the meniscus can buffer it through deformation to protection the knee cartilage [2]. The integrity of function and structure of meniscus is the premise to ensure the health of knee cartilage. However, meniscectomy is mainly the first option when the injury occurs in the medial area of the meniscus because the vessels only exist in the peripheral area [3]. Some previous studies found that total/partial meniscectomy could reduce the contact area between meniscus and articular cartilage, leading to the increased peak contact pressure between meniscus and articular cartilage [4–6]. Exorbitant stress on cartilage causes subsequent degenerative changes, severe pain, and dysfunction. To overcome these shortcomings, the meniscal allograft transplantation has been regarded as a promising substitution for meniscectomy. Basic daily activities can be improved in the short or medium term after meniscal replacement. However, most allografts may atrophy and undergo collagen remodeling after long-term transplantation, which may affect the mechanical strength and lead to graft tear, joint instability, and degenerative injury [7].

Tissue engineering strategies are emerging as a possible solution to repair or replace the injured or defected meniscus. Numerous biomaterials, such as synthetic polymers, hydrogels and tissue-derived materials have been investigated. Among them, poly(ε-caprolactone) (PCL), polyurethane (PU) polymers and collagen-based materials have been widely used in meniscus reconstruction. Chang et al. designed a PCL scaffold loaded with connective tissue growth factor (CTGF) and transforming growth factor-β3 (TGF-β3) to promote the collagen production on the peripheral and medial areas of the meniscus [8]. Some scholars have utilized meniscal ECM as a composite scaffold, including PCL/GelMA/ECM hybrid scaffold or PCL/PU/ECM hybrid scaffold [9, 10]. Artificial meniscal prosthesis is made of non-absorbable polycarbonate polyurethane (PCU) elastomers, which can avoid the above-mentioned risks and approved by US Food and Drug Administration (FDA). The commercial PCU artificial meniscus is 100% filled and designed to be oval to cover the cartilage surface. However, this shape is unbeneficial for improving the load transmission inside the joint cavity. The difference in elastic modulus between PCU and natural meniscus is not advantageous for improving the peak pressure applied to the surface of articular cartilage, resulting in the pathological symptoms of articular cartilage after meniscectomy [11, 12].

Pore size, porosity and pore interconnectivity are important factors for cell adhesion and proliferation. Meanwhile, the biomechanical strength should also be considered for meniscal scaffolds to bear femur/tibia compressive load [13]*.* Meniscus scaffolds with different mesh geometries have been attempted. Chen et al. constructed a wedge-shaped porous PCL scaffolds with circumferentially and radially oriented fibers as a backbone, followed by injection with meniscus extracellular matrix (MECM)-based hydrogel. The hybrid scaffold showed favorable biomechanical properties and successfully promote whole meniscus regeneration [14]. Zhang et al. also created a wedge-shaped ring scaffold of PCL seeded with marrow-derived mesenchymal stem cells (MSCs). Biomechanical and biochemical stimuli were applied to induce zonal expression of collagens [15]. Abar et al. 3D printed porous PCU scaffold in a crosshatch pattern with different pore size to match the stiffness of native weight-bearing soft tissues [16]. However, the mechanical diversity between these hybrid scaffolds and natural meniscus is still noticeable, and the recovery of the function of meniscus is a challenge. Thus it is necessary to optimize the scaffold structure to improve the mechanical properties, in order to mimic the natural meniscus.

Traditional design of meniscal scaffolds is mostly on the basis of a rod connected porous structure, which is easy to fabricate and cooperate with growth factors or other bioactive materials. However, this type of grid scaffolds cannot mimic the mechanical characteristics of natural meniscus, and improvement of the porous structure is essential. Triply periodic minimal surface (TPMS) method based scaffolds seem promising owing to their excellent interconnectivity and high surface to volume ratio. In fact, this mathematical modeling method provides a precise control over internal architectures and complex external anatomical shapes. Compared to traditional grid structures, the TPMS structure has led to less stress concentrations [17]. Meanwhile, the TPMS scaffolds can affect the cellular behavior though the surface curvature. For instance, vascularization is more likely to occur on the surface with a small curvature [18], and the arrangement of fiber tissue is easily regulated by the surface curvature [19]. In our previous research, we proposed a deformed Primitive TPMS structure with ellipse shape unit resulting from larger numbers of unit cell repetitions in the load bearing direction. Mechanical test proved that this structure can facilitate good transfer of load to reduce the stress concentration area, stress extremes and extrusion displacement of meniscus, thus protecting the articular cartilage [20]. However, in vivo studies have not been conducted to evaluate the TPMS meniscal scaffolds outcomes. The influence of TPMS structure on cell distribution, migration and proliferation should also be discussed.

Therefore, the overall goal of this study was to develop a meniscal scaffold both mechanically and biologically mimic those properties of the native meniscus. First of all, meniscus scaffold using deformed primitive TPMS structure was designed. The effects of load transmission on different meniscal scaffolds were evaluated by the finite element analysis (FEA) in a three-dimensional (3D) human knee model. The porous PCU scaffold was 3D printed and then implanted into swine knee joints to evaluate the meniscus regeneration potential according to medical imageology, histological, and mechanical assessments. We supposed that TPMS-based PCU meniscal scaffolds could be advantageous for improving the therapeutic efficacy for patients with meniscal tears.

## Materials and methods

### Data acquisition and 3D reconstruction of natural meniscus and knee joint

A 35-year-old male volunteer without any symptoms of osteoarthritis or meniscal tears was scanned by a 3 T magnetic resonance (MR) scanner (uMR 770; United Imaging Co., Ltd., Shanghai, China) and a GE Lightspeed 16-slice computed tomography (CT) scanner (GE Healthcare, Chicago, IL, USA). During the scanning, the volunteer kept the supine position with a maker on the lower limb to fit the coordination of the two scanning systems. For the MR imaging (MRI), the extended echo train sequence was performed with a slice thickness of 1.5 mm and a field of view (FOV) of 152 mm. For the CT scanning, the slice thickness was 0.625 mm, and the FOV was 500 mm.

The images saved in digital imaging and communications in medicine (DICOM) format were imported into the MIMICS 19.0 software (Materialise, Leuven, Belgium) to complete the 3D reconstruction. The bone objectives were reconstructed using the segmentation of bone structures from CT images, and the cartilage, meniscus, and ligaments were manually segmented from the MR images under the supervision of an experienced radiologist and an experienced orthopedist.

### Design and fabrication of the TPMS-based porous meniscal scaffold

The deformed triply periodic primitive porous structure can be generated by finding the *Φ* = 0 isosurface of the TPMS equations. This surface is the boundary between solid- and void-material phases [21].

1 $$\phi_{{\text{P}}} (x,y,z) = \cos (k_{x} x) + \cos (2\pi k_{y} y) + \cos (k_{z} z) - t$$

In Eq. (1), *k*_*i*_ represents the functional periodicity of the TPMS, which was defined as

2 $${k}_{i}=2\pi \frac{{n}_{i}}{{L}_{i}}\left(\mathrm{with} i=x, y, z\right)$$

where *n*_*i*_ indicates the times of unit repetitions in the tri-axial directions, *L*_*i*_ is the absolute size of the unit in those directions, and *t* determines the volume fraction *ρ* of the porous structure.

The modeling process was performed using Wolfram Mathematica 11.0 software (Wolfram Inc., Champaign, IL, USA) through importing the above-mentioned equations. The 3D models were generated by the “RegionPlot3D” function. Scaffolds with different volume fractions and functional periodicities were designed in the present study. The morphological parameters of the scaffolds are listed in Table 1. The scaffolds used for mechanical compression were exported as cubic with a bottom edge of 10 mm and a height of 20 mm. The scaffolds used for FEA and in vivo applications were established by the Boolean function in Magics 19.0 software (Materialise) with the natural meniscal model obtained through MR images. Finally, these models were exported as STL files for the future 3D printing and FEA.

**Table 1:** Morphological parameters of the scaffold

	Volume fraction *ρ* (%)	Function periodicity *k*_*x*_	Function periodicity *k*_*y*_	Function periodicity *k*_*z*_
Pvol	36.70	25π	25π	50π
Pfun	41.80	35π	35π	70π

The models for mechanical test and in vivo study were fabricated through the 3D printing technique using the Bio-Architect® WS 3D printer (Regenovo Co., Ltd., Shanghai, China). The FDA-approved PCU particles (ChronoFlex C 80A; AdvanSource Biomaterials Inc., New York, NY, USA) were filled in the copper barrel and printed with a 22G copper nozzle. The temperature of printing was set to 195 ℃, the temperature of platform was set to 10 ℃, the air pressure was set to 0.35 MPa, the speed of printing was set to 10 mm/s, and the distance between filaments was set to 1000 and 600 μm for the grid scaffold and TPMS-based scaffold, respectively.

### FEA

The FEA was performed using the Abaqus 2017 software (SIMULIA Inc., Rhode Island, USA). Briefly, the STL files were re-meshed by 3-matic 11.0 software (Materialise). The ligaments were considered as nonlinear materials, and modeled as transversely isotropic nearly-incompressible neo-Hookean materials [22, 23] using the strain-energy function as follows:

$$\Phi ={C}_{1}\left(\overline{{G }_{1}}-3\right)+\frac{1}{{D}_{1}}{({J}_{F}-1)}^{2}+S(\lambda )$$

*S(λ)* represents the strain-energy function of the fiber family, which could be satisfied with the following conditions:

$$\uplambda \frac{dS}{d\lambda }=\left\{\begin{array}{c}0, \lambda \le 1\\ {C}_{3}\left({e}^{\left(\lambda -1\right){C}_{4}}-1\right), 1<\lambda <{\lambda }^{*} \\ {C}_{5}\lambda +{C}_{6}, \lambda \ge {\lambda }^{*}\end{array}\right.$$

where *C*_*1*_ is a bulk material constant related to the shear modulus μ (C_1_ = 2/μ); *J*_*F*_ represents the Jacobian matrix of the deformation gradient F; $\overline{{G }_{1}}$ denotes the first invariant of the left Cauchy-Green tensor $\overline{{G }_{1}}=tr{\overline{FF} }^{T}$ with the modified deformation gradient $\overline{F }$ ($\overline{F }={J}_{F}^{-0.33}F$). The values of the material constants C_1_, C_3_, C_4_, C_5_, and D_1_, are listed in Table S1.

The bone objectives were regarded as linear materials with an elastic modulus (E) of 7,300 MPa and a Poisson’s ratio (ν) of 0.3 [24]. The articular cartilage and the menisci were both regarded as the single-phase linear elastic and isotropic materials. The average material properties for the tissues were E = 15 MPa, ν = 0.475, and E = 120 MPa, ν = 0.45, respectively [25–27]. The PCU meniscal scaffold was regarded as isotropic neo-Hookean materials with a stiffness of 11 MPa and a Poisson’s ratio of 0.49 [28].

During the simulation process, the ligaments and the bones were rigidly fixed, and the kinematic constrain was set between other subjects, such as bone, cartilage, and meniscus. A vertical compressive load of 1150 N was applied to simulate the balanced standing based on previous studies [29, 30]. The tibia and fibula were fixed in all translational and rotational directions. The femur was unconstrained in all translational and rotational degrees of freedom at 0° of flexion. Five types of meniscus were employed in the FEA, including native meniscus, solid PCU scaffold, grid PCU scaffold, Pvol and Pfun TPMS-based scaffolds. In addition, the cubic scaffolds made up of gird, Pvol, and Pfun units were analyzed under the force of 100 N applied to the top surface and fixation at the bottom to evaluate the stress conduction behavior inside the scaffolds.

### Mechanical tests

The mechanical tests were carried out using a universal testing machine (E3000; Instron, Norwood, MA, USA) equipped with a 3 kN load cell. The tests were performed on three types of scaffolds (grid, Pvol, and Pfun) and repeated for three times. The compressive stress–strain test was carried out under the compression speed of 2 mm/min. For the multi-cycle compression test, the sample was compressed to the strain of 30%, and then, restored to the initial height for 9 consecutive cycles. For the stress-relax test, the sample was quickly preloaded for the strain of 10% that maintained for 300 s, and the test was repeated for five times until the strain reached 50%. For the tensile test, the sample was cracked under the tensile speed of 2 mm/min. The Young’s modulus comprised the approximated linear fitting values under the strain deformation of 35–40%. The toughness was calculated from the area below the compressive stress–strain curve until fracture.

### In vivo study

A total of 15 male Bama mini pigs with a bodyweight of about 25 kg were utilized in the study and randomly divided into 3 groups (*n* = 5 pigs for each group). The anesthesia procedure was performed using lidocaine and propofol. A 6 cm long transverse incision was made on the outside of the knee joint. The lateral meniscus was exposed after incision of muscle and joint capsule. After the lateral meniscectomy, the control group remained blank, a grid PCU scaffold was implanted in the grid group, and a TPMS-based PCU scaffold was implanted in the TPMS group. Then, the incision was closed layer by layer, and cefuroxime sodium was injected intramuscularly for 3 days after surgery to avoid infection. All animals were sacrificed at week 12 post-operation to evaluate the effects on meniscal regeneration and cartilage protection of these scaffolds.

### Evaluation of the meniscal regeneration and cartilage protection

The knee joint was scanned using the 9.4 T Bruker Biospec 94/20 USR Micro-MRI system (Bruker, Bremen, Germany) with the fat-suppressed proton–density weighted turbo spin-echo sequences. The FOV was 65 mm, the echo time was 6.28 ms, the repetition time was 1590 ms, and the slice thickness was 1 mm. The micro-CT analysis was performed using the VivaCT-80 system (SCANCO Medical AG, Brüttisellen, Switzerland) at voltage of 70 kV and current of 114 μA, with the FOV of 31.9 mm and a voxel size of 15.6 μm. The bone volume to total volume ratio (BV/TV), trabecular thickness (Tb.Th), trabecular number (Tb.N), and trabecular separation (Tb.Sp) were obtained through the same system.

The macroscopic view of the femoral and tibial cartilage was scored according to the International Cartilage Repair Society (ICRS) macroscopic scoring system. The scoring level was dependent on the color, integrity, contour, and smoothness of the cartilage surface. The detailed scoring system is presented in Table S2.

Five regions for each group were enrolled in the histological evaluation, containing femoral cartilage, tibial cartilage, meniscal anterior horn, meniscal body, and meniscal posterior horn. The histological sections stained with hematoxylin & eosin (H&E), toluidine blue, Safranin O, and collagen I were used to assess the status of cartilage according to the O'Driscoll scoring system. In addition, three slices of Safranin O staining from each group were randomly selected for the semiquantitative analysis of the GAG content using the imageJ 1.53e software (NIH, USA). All the sections were observed using a BX-53 microscope (Olympus, Tokyo, Japan). The detailed scoring system is summarized in Table S3.

To further investigate the collagen content in the regenerated meniscus, the immunofluorescence of collagen I and collagen II was used with the primary antibody of Anti -Collagen I Rabbit pAb (GB11022-3, Servicebio, China) and Anti -Collagen II Mouse mAb (GB12021, Servicebio, China), and the secondary antibodies of Alexa Fluor® 488-conjugated Goat Anti-Rabbit IgG (GB25303, Servicebio, China) and Cy3 conjugated Goat Anti-mouse IgG (GB21301, Servicebio, China). The stained slices were scanned using the fluorescence microscope (Nikon, Ti-U). The fluorescence intensity was analyzed using the imageJ 1.53e software to achieve the semiquantitative results of collagen content.

The mechanical tests, including compression crack test, multi-cycle compression test, and tensile test were performed on different regions of the regenerated meniscus. Briefly, six regions from the meniscus were used in the mechanical test according to a previous study [31], including the bulk tensile group, the radial direction tensile group, the inner tensile group, the outer tensile group, the inner compression group, and the outer compression group (Figure S1). For the tensile test, the sample was rectangular in shape with a thickness of 1 mm and a length of 3 mm. For the compression test, the sample was cylinder in shape with a diameter of 2 mm and a thickness of 1 mm. Three samples for each region were employed in this test.

### Statistical analysis

Statistical analysis and exponential curve fitting were performed using SPSS 19.0 software (IBM Corp., Armonk, NY, USA) and IGOR Pro 6.12 software (WaveMetrics Inc., Portland, OR, USA). The data were presented as mean ± standard deviation (SD), and evaluated by an unpaired Student’s t-test. *P* < 0.05 was considered statistically significant.

## Results

### 3D models of the knee joint and the porous meniscal scaffolds

**Fig. 1:**
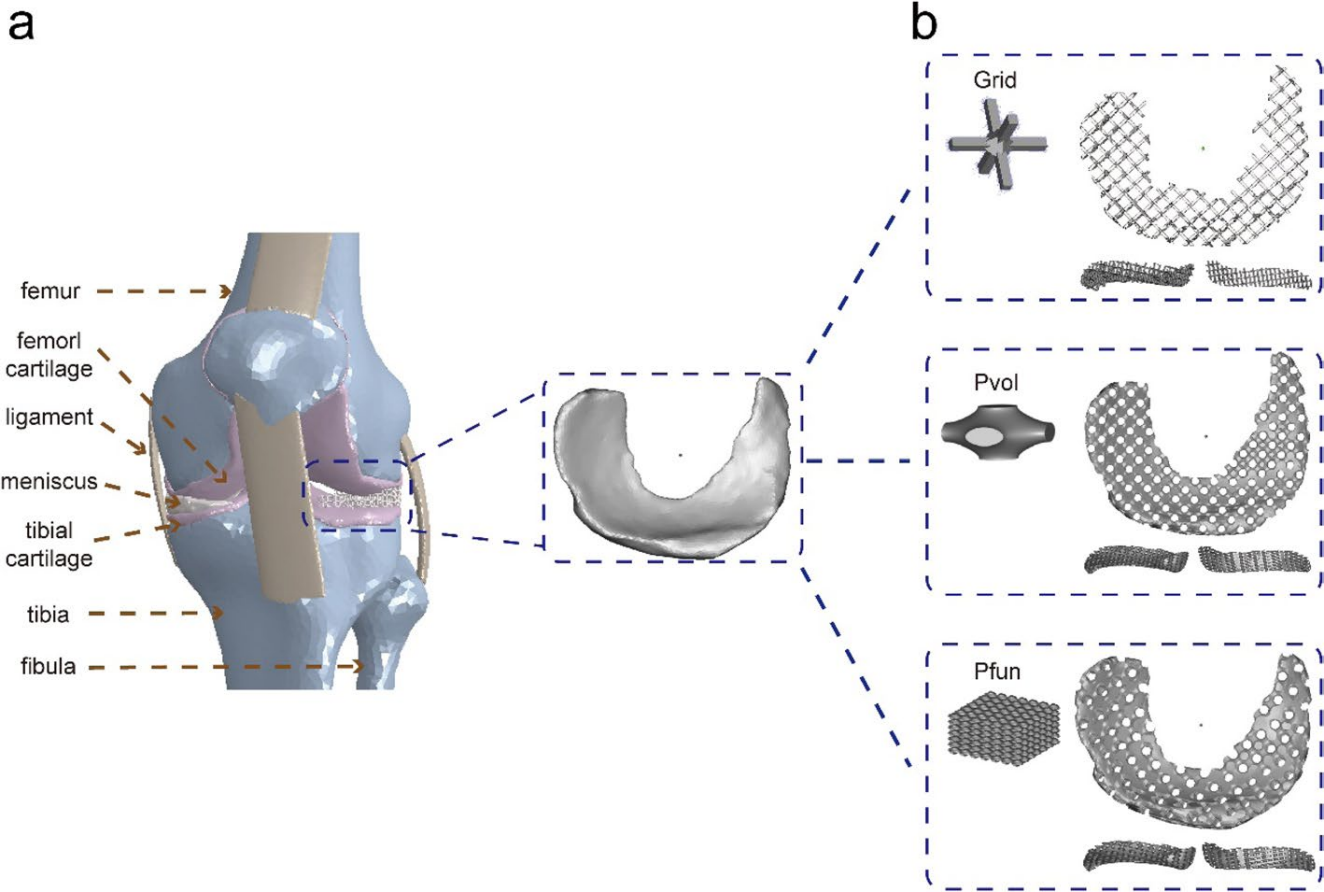
The diagram of FEA model and porous meniscal scaffold. **a** The 3D knee model used in the FEA. **b** Three porous meniscal scaffolds were used in the FEA

As shown in Fig. 1a, the 3D model contained main objectives of the knee joint, including the bony structures, cartilage, menisci, and ligaments. Other soft tissues, such as the muscle, fascia, and skin were not considered in the model. The morphology of the lateral meniscus was employed to assess different pore structure units, defined as grid, Pvol, and Pfun (Fig. 1b), and the models for in vitro compression test are shown in Figure S2. Compared with Pvol, Pfun obtained a larger volume fraction and a longer functional periodicity. The porosity and the surface area of the same-sized solid structure were defined as 0% and 100%, respectively. The detailed structural characteristics of the three scaffolds are listed in Table 2.

**Table 2:** Structure characteristics of the porous scaffolds

	Porosity (%)	Surface area (%)	Pore size (μm)	Distance between pores (μm)
Solid	0	100	/	/
Grid	89.53	121.52	1500	400
Pvol	63.30	229.67	1200	800
Pfun	58.20	197.28	1400	1200

### The effects of porous meniscal scaffolds on stress conduction assessed by FEA

To assess the influences of scaffolds made up of different porous units under stress simulation, the FEA of cubic scaffold and total knee joint was performed. The results revealed that TPMS-based units could improve the stress conduction behavior. As illustrated in Figure S3, under the compression of the same force, the Pvol and Pfun scaffolds demonstrated a better load buffering capability. The stress applied to the grid scaffold was an order of magnitude higher than that applied to the Pvol and Pfun scaffolds. When the threshold value was set to 60 MPa, a large number of red areas and a small amount of gray areas could be observed on the rod parallel to the stress direction, and the peak stress was more than 90 MPa (Figure S3a). However, the peak stress applied to Pvol and Pfun scaffolds was lower than 10 MPa. For Pvol scaffold, the main color on the rod parallel to the stress direction was green, with a small amount of yellow and red areas when the threshold value was set to 4 MPa (Figure S3b). However, the color of the same region was entirely green in the Pfun scaffold under the same threshold value (Figure S3c). The results indicated that the average stress applied to Pvol scaffold was higher than that applied to Pfun scaffold.

**Fig. 2:**
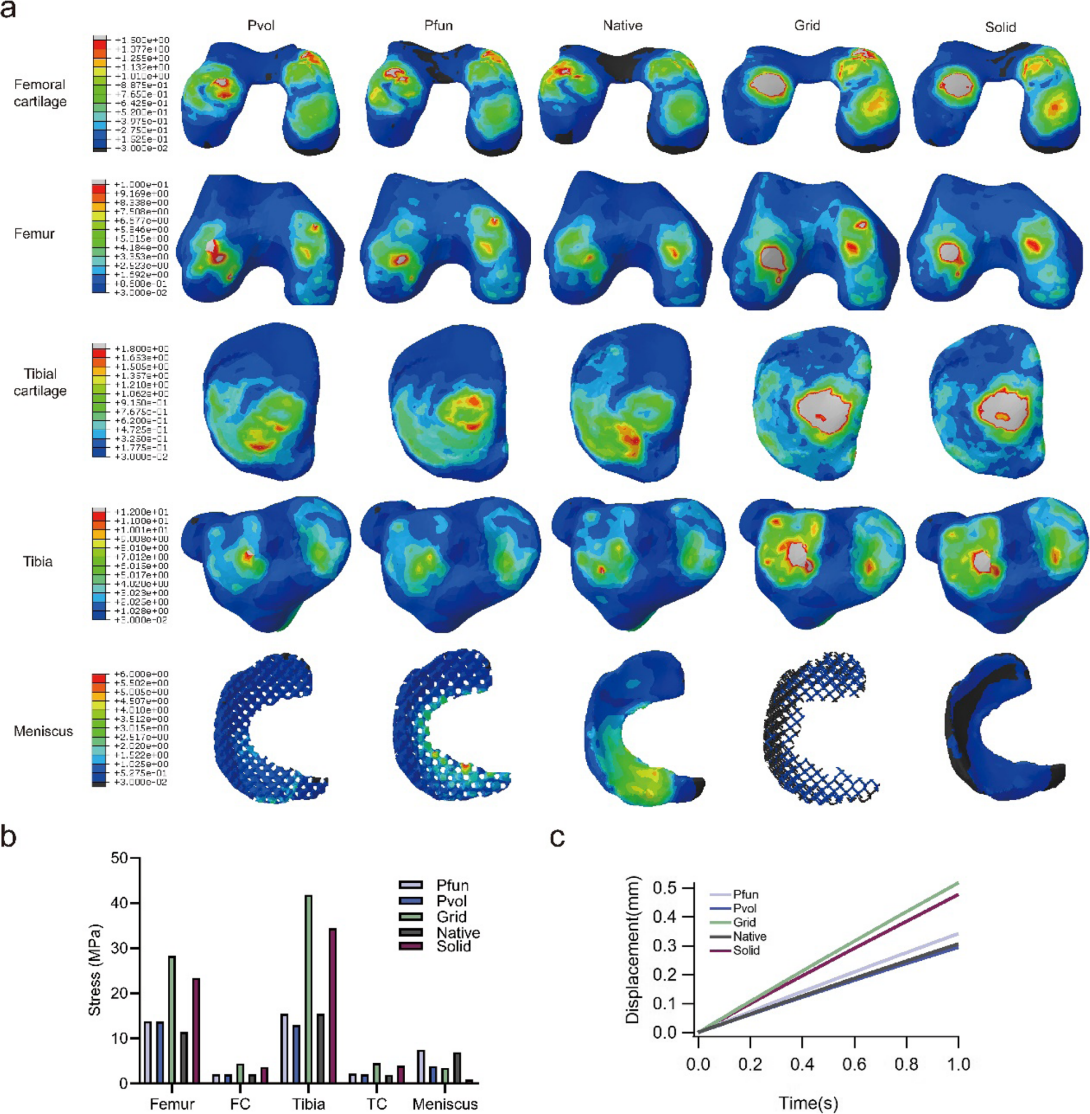
The results of the FEA. **a** The stress nephogram on bone, cartilage, and meniscus in the five groups. The color from deep blue to red represented the stress changing from small to large, the gray represented the stress exceeding the threshold in the legend. **b** The peak stress applied to the main objects of the knee model. **c** The displacement of meniscal extrusion

The FEA results of knee joint are displayed in Fig. 2. A large area of gray color can be found on all the four objectives (femoral cartilage, femur, tibia, and tibial cartilage) in grid and solid scaffold groups (Fig. 2a), indicating that the grid and solid meniscal scaffolds were unbeneficial for buffering the load in knee joint. The femur in the Pvol group showed a more obvious stress concentration than that in the Pfun group, and stress distribution area on tibial cartilage was similar to the native group. Compared with Pvol scaffold, the higher similarity in stress distribution on femoral cartilage and tibia could be observed in the Pfun group compared with that in the native group. Moreover, the stress distribution on Pfun meniscal scaffold was the most similar to the native meniscus among all the groups.

The peak stress applied to these objectives could directly demonstrate the diversity among these groups (Fig. 2b). The grid and solid groups had a significantly higher stress in the bony structures and cartilage. Meanwhile, stress applied to meniscal scaffolds in these two groups was low, especially in the solid group. This phenomenon represented that the solid PCU meniscal scaffolds were almost impossible to buffer the stress between two articular surfaces. In the Pfun group, the peak stress in the femoral cartilage, tibia, tibial cartilage, and meniscus was as same as that in the native group. In the Pvol group, the peak stress in the femoral cartilage and tibial cartilage was as same as that in the native group. The numerical values of the peak stress are listed in Table S4.

The meniscal extrusion displacement is shown in Fig. 2c, in which the grid and solid scaffolds exhibited a larger displacement than that in the other three groups, and the Pvol scaffold was the most similar to that of the native meniscus among all the groups.

### Mechanical behaviors of different porous meniscal scaffolds

**Fig. 3:**
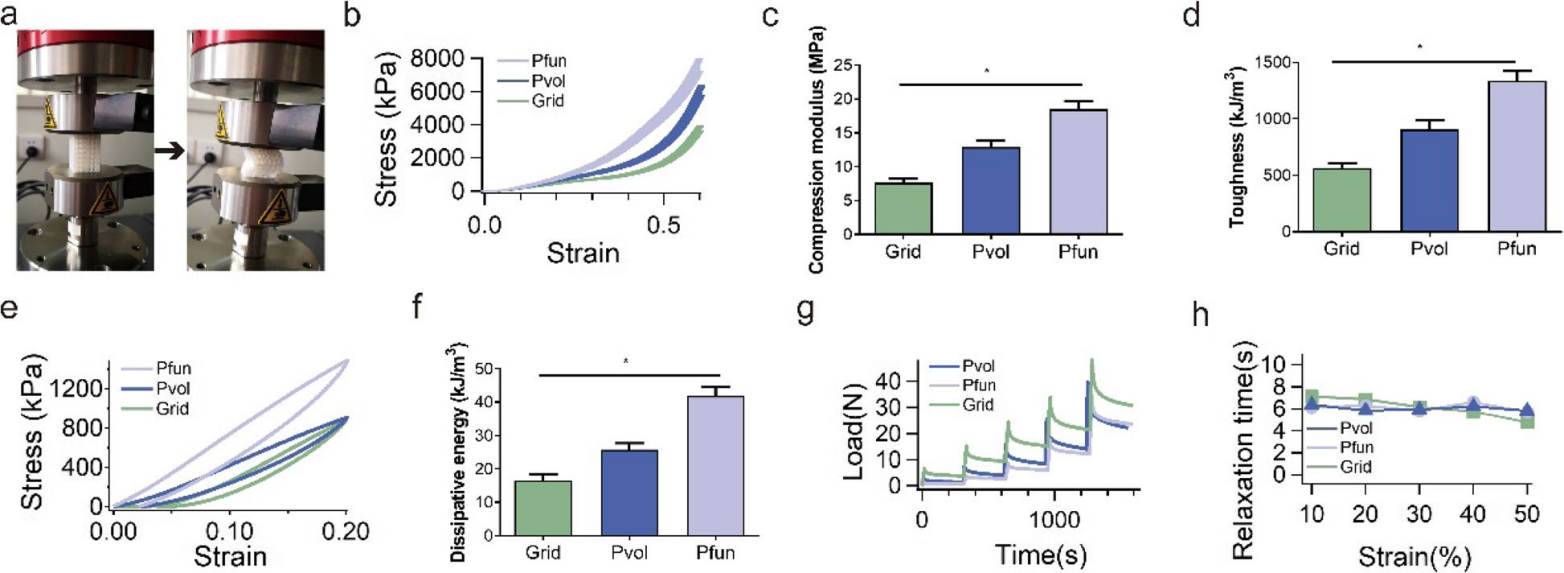
The results of the in vitro compression tests. **a** The process of the compression test. **b** The stress–strain curve of the compression test. **c** The compression modulus in the three scaffolds. **d** The toughness in the three scaffolds. **e** The stress–strain curve of the single-cycle compression at 20% strain. **f** The dissipative energy for the hysteresis curve. **g** The curve of the stress-relaxation test. **h** The relaxation time scale in the three scaffolds. All the tests were repeated for 3 times

The diagram of in vitro mechanical tests is illustrated in Fig. 3a. To evaluate the mechanical behaviors of grid, Pvol, and Pfun scaffolds, compressive stress–strain test, single-cycle compression test of 20%, and stress-relaxation test were performed. The stress–strain curve is shown in Fig. 3b, in which the limit stress of 60% exhibited a significant difference among the three groups. Therefore, the compression modulus (Fig. 3c) and the toughness (Fig. 3d) demonstrated the same tendency, and the Pfun scaffold obtained the largest compression modulus and toughness. The single-cycle compression curve is displayed in Fig. 3e, and the dissipative energy of each cycle is presented in Fig. 3f, in which the Pfun scaffold also gained the largest compression limit and energy dissipation. Compared with the Pvol and grid scaffolds, the hysteresis in the Pfun scaffold was notably obvious, and the hysteresis in the grid scaffold was relatively insignificant, which represented that more energy was dissipated from mechanical to heat. The numerical values of the above-mentioned results are listed in Table 3. The stress-relaxation curve is shown in Fig. 3g, and the stress-relaxation behavior can be observed in all the three types of porous scaffolds. The characteristic relaxation time scales at different strains are presented in Fig. 3h, in which the two TPMS-based scaffolds had a similar relaxation time, while the relaxation time decreased in the grid scaffold.

**Table 3:** Mechanical properties of the porous scaffolds

	Compression modulus (MPa)	Toughness (kJ/m^3^)	Limit compression stress (MPa)	Energy dissipation (kJ/m^3^)
Grid	7.63 ± 0.59	566.91 ± 35.69	3.77	16.56 ± 1.75
Pvol	12.90 ± 0.97	907.18 ± 84.04	6.05	25.76 ± 1.92
Pfun	18.48 ± 1.14	1335.29 ± 90.69	7.59	41.87 ± 2.59

### In vivo evaluation of the porous meniscal scaffolds

Considering the FEA results and mechanical behaviors, the Pfun scaffold was chosen to be implanted in the animal model as the representative of TPMS porous structure. Thus, the in vivo study included the blank group (excision of lateral meniscus), the grid group (implantation of grid scaffold), and the TPMS group (implantation of Pfun scaffold) (Figure S4a). The implantation process is shown in Figure S4b, and the two types of scaffolds were covered and filled with regenerative tissue after 12 weeks of implantation (Figure S4c).

**Fig. 4:**
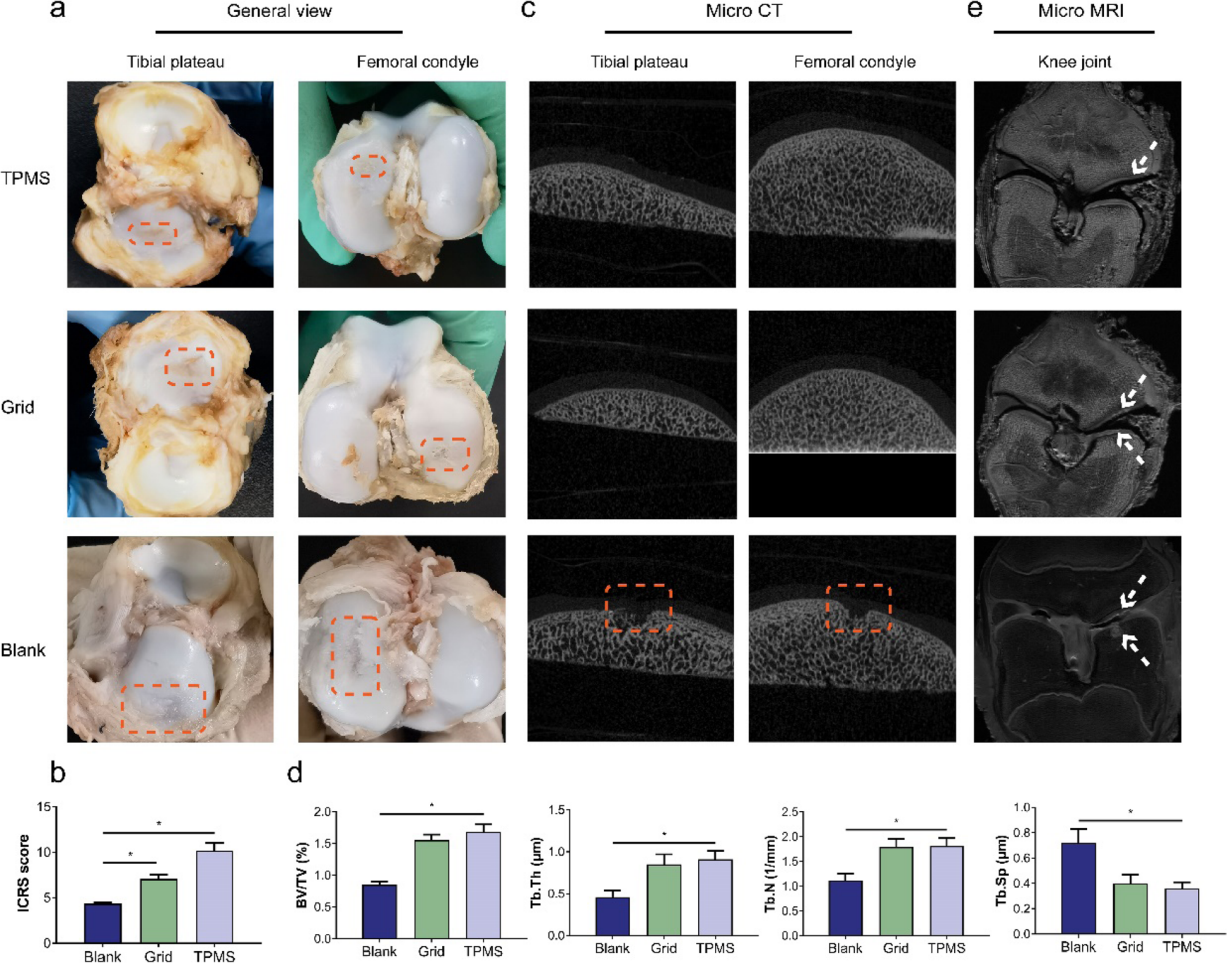
The general view and radiographic results of the knee joint after 3 months of implantation, *n* = 5. **a** General view of the tibial plateau and femoral condyle, the square indicated the lesion cartilage of each group. **b** The ICRS scores in the three groups, *P < 0.05. **c** The micro-CT scan images in the three groups, the square indicated the lesion region of subchondral bone in the blank group. **d** The trabecular parameters in the three groups, *P < 0.05. **e** The micro-MR images in the three groups, the arrows indicated the cartilage injury in the TPMS and grid group, and the cartilage and subchondral bone defect in the blank group

The general views of the articular surface for the three groups are exhibited in Fig. 4a, in which different degrees of lesions can be observed on the tibial plateau and femoral condyle for all the groups (marked with red box). The TPMS group only demonstrated a slight degeneration on the cartilage surface, as the grid group presented an obvious cartilage damage. The blank group had the most serious situation, in which severe cartilage destruction was found in both the upper and lower articular surfaces. Therefore, the TPMS group obtained the highest ICRS score, and a significant difference could be found among the three groups (Fig. 4b). According to the micro-CT scan, the subchondral bone in the TPMS and gird groups was normal, while a notable bone loss could be found in the blank group (marked with red box, Fig. 4c). For the trabecular parameters achieved by micro-CT scan, the blank group presented a statistically significant difference, including lower values of BV/TV, Tb.Th and Tb. N, and a higher Tb.Sp value (Fig. 4d).

Based on the micro-MR images, the porous meniscal scaffolds were clearly demonstrated, their position was normal, and a small amount of effusion was found around the grid scaffold (Fig. 4e). The cartilage and bone pathological changes could also be observed in these groups. In the TPMS group, only a slightly blurred margin was existed at the cartilage region, indicating that the cartilage degeneration occurred at the cartilage surface. In the grid group, an obviously irregular high brightness area could be found at the cartilage region, demonstrating the cartilage matrix damage. In the blank group, the high brightness area appeared at both cartilage and subchondral bone regions, which revealed the cystic change in bony structure and cartilage matrix damage.

### Histological analysis of the in vivo study

**Fig. 5:**
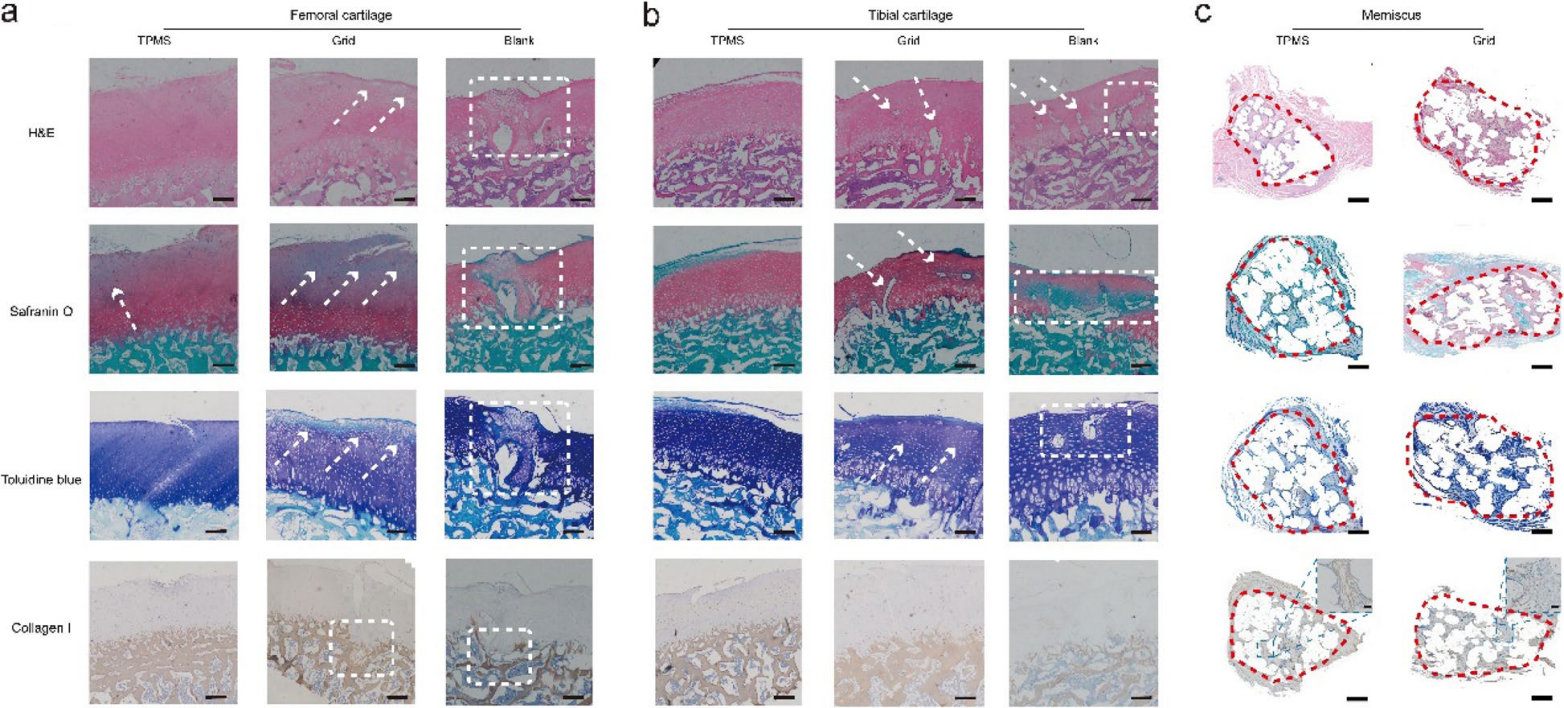
Histological results of the cartilage and meniscus, *n* = 5. **a** The histological staining of femoral cartilage in the three groups, the arrows indicated the poorly recovered region at cartilage layer, the squares indicated the obviously defect region at cartilage and subchondral bone, scale bar: 500 μm. **b** The histological staining of tibial cartilage in the three groups, the arrows indicated the poorly recovered region at cartilage layer, the squares indicated the obviously defect region at cartilage and subchondral bone, scale bar: 500 μm. **c** The histological staining of meniscus in the three groups, the regenerated region were indicated by the square, scale bar: 1000 μm

To evaluate the cartilage protection effect of the porous scaffolds, several staining methods were performed on the paraffin-embedded sections of healthy cartilage and three experimental groups. The TPMS group exhibited the best performance in cartilage protection among the three groups. The H&E staining apparently showed that the cellular arrangement and structural integrity in the TPMS group (Fig. 5a, indicated by arrows and squares) were similar to those in the healthy cartilage (Figure S5a). In the grid group, the irregular holes could be observed and the cellular arrangement was abnormal. Meanwhile, a noticeable ECM loss and structural damage could be found in the blank group. For Safranin O staining, the red dye at the surface became light in the TPMS group, which indicated the mild loss of glycosaminoglycan (GAG) in the ECM. In the grid group, the absence of red dye ECM was more obvious and a crack was existed, which indicated more serious GAG loss and cartilage damage. The semi quantitative analysis shown that the native cartilage had the highest GAG content (Figure S6a). and the TPMS group also obtained large amount of GAG, which were notably higher than gird and blank groups. Similar to the H&E staining, the blank group also presented a severe damage in ECM. The same findings could be found in the toluidine blue staining. A relatively normal chondrocyte arrangement and ECM dye were noted in the TPMS group, the grid group showed noticeable cartilage degeneration and injury, and cartilage in the blank group was already injured. The collagen I staining indicated the status of bony structure, and the TPMS and grid groups had a relatively complete trabecular structure. Although some damage occurred at the grid group, the trabecular density and thickness were similar to the intact subchondral bone. However, the brown dye was apparently reduced in the blank group, which represented the loss of bony structure.

The same staining results could be observed in the tibial cartilage (Fig. 5b, indicated by arrows and squares). Compared with the healthy tibial cartilage (Figure S5b, S6B), the TPMS group demonstrated the most similar cellular arrangement, cartilage ECM dye, higher GAG content, and morphology of bony structure among these groups. According to the performance in the histological assessment, the TPMS group obtained the highest score in the O’Driscoll scoring system, which was significantly higher than that in the other groups (Figure S6c).

The histological staining results of meniscus are exhibited in Fig. 5c. Compared with the native meniscus (Figure S5c), the content of GAG in the grid scaffold was more obvious, red and blue dyes could be observed in the pores of the scaffolds, and these colors were deeper than those in the native meniscus. The staining of the TPMS-based scaffold was similar to the native meniscus, no red dye was existed in the outer region, and the blue dye was light. The collagen I staining of the TPMS-based scaffold was more explicit than the grid scaffold. According to the magnified images of the two scaffolds, a large amount of the regenerated tissue in the TPMS-based scaffold was brown dye, while the newly born tissue in the grid scaffold was blue stained. The results of immunofluorescence can obviously prove the results. The TPMS group exhibited stronger green fluorescence and weaker red fluorescence at the pores of the scaffolds (Figure S7a), which were similar to the tendency of native meniscus. The semi quantitative analysis of fluorescence intensity clearly demonstrated that the native meniscus had the highest relative content of collagen I than the two kinds of scaffolds (Figure S7b), and the significant difference was existence between TPMS and grid groups. The relative content of collagen II shown the opposite tendency (Figure S7c), the grid group had the highest volume, and the TPMS group was slightly higher than the native meniscus. The magnification images of the immunofluorescence staining results were shown in Figure S8.

### Mechanical properties of the porous scaffolds with regenerated meniscus

To comprehensively assess the function of the regenerated meniscus, the compressive and tensile tests were performed on six regions of the meniscus. The native meniscus had the largest modulus in both the inner and outer regions, whereas the TPMS-based scaffold exhibited the best performance in terms of energy dissipation and deformation recovery.

**Fig. 6:**
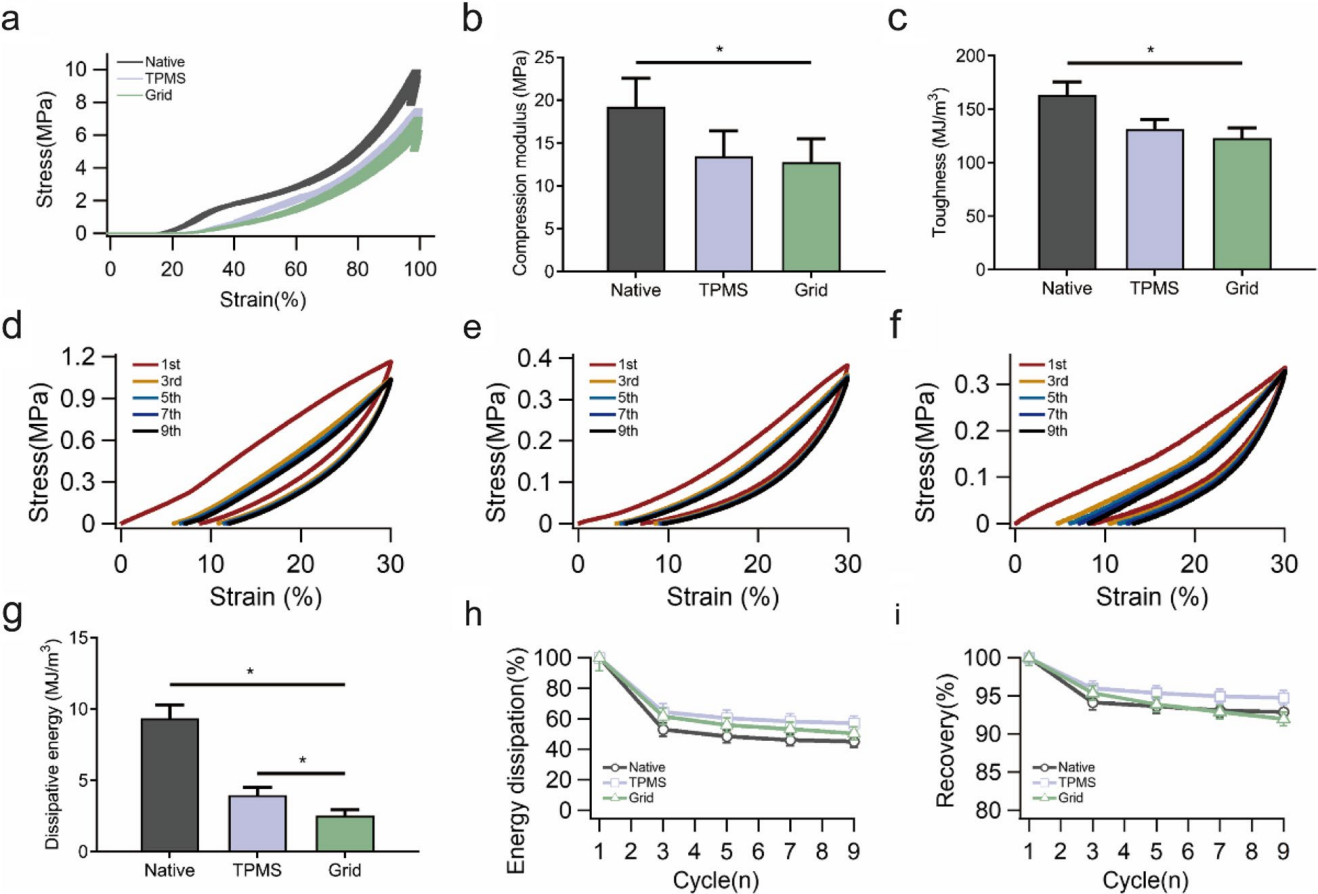
The results of the compression tests in the inner region, n = 3. **a** The stress–strain curve of the compression test. **b** The compression modulus in the three scaffolds, *P < 0.05. **c** The toughness in the three scaffolds, *P < 0.05. **d** Five compression-relaxation cycles in the native meniscus. **e** Five compression-relaxation cycles in the TPMS group. **f** Five compression-relaxation cycles in the grid group. **g** The dissipative energy of the 1st cycle in three groups, *P < 0.05. **h** The percentage of dissipated energy in nine continuous compression-relaxation cycles. **i** The recovery percentage in nine continuous compression-relaxation cycles

The compression testing of the inner region is shown in Fig. 6, including compressive stress–strain test and multi-cycle compression test. The native meniscus obtained the largest compressive limit stress at 9.75 MPa (Fig. 6a), and the compression modulus was more than 19 MPa (Fig. 6b). Thus, the toughness of native meniscus was also the highest (Fig. 6c). The TPMS and grip groups had similar numerical values in compression testing of inner region. A more obvious diversity could be found in the multi-cycle compression test. The hysteresis and energy dissipation were existed in all the three groups (Figs. 6d-f), and the stress–strain curve of each single-cycle can clearly illustrate this phenomenon (Figures S9a-c). The native meniscus had the largest dissipative energy in the first cycle (Fig. 6g), and there were statistically significant differences between each pair of groups. However, all the groups demonstrated a notable reduction in dissipative energy in the subsequent cycles. The TPMS group maintained about 57% of the energy after 10 cycles, which remained 7% and 12% higher than grid group and native meniscus, respectively (Fig. 6h). The TPMS group maintained the minimum deformation loss at 5.27%, which reduced by 1.86% and 2.76% versus native meniscus and grid group (Fig. 6i).

**Fig. 7:**
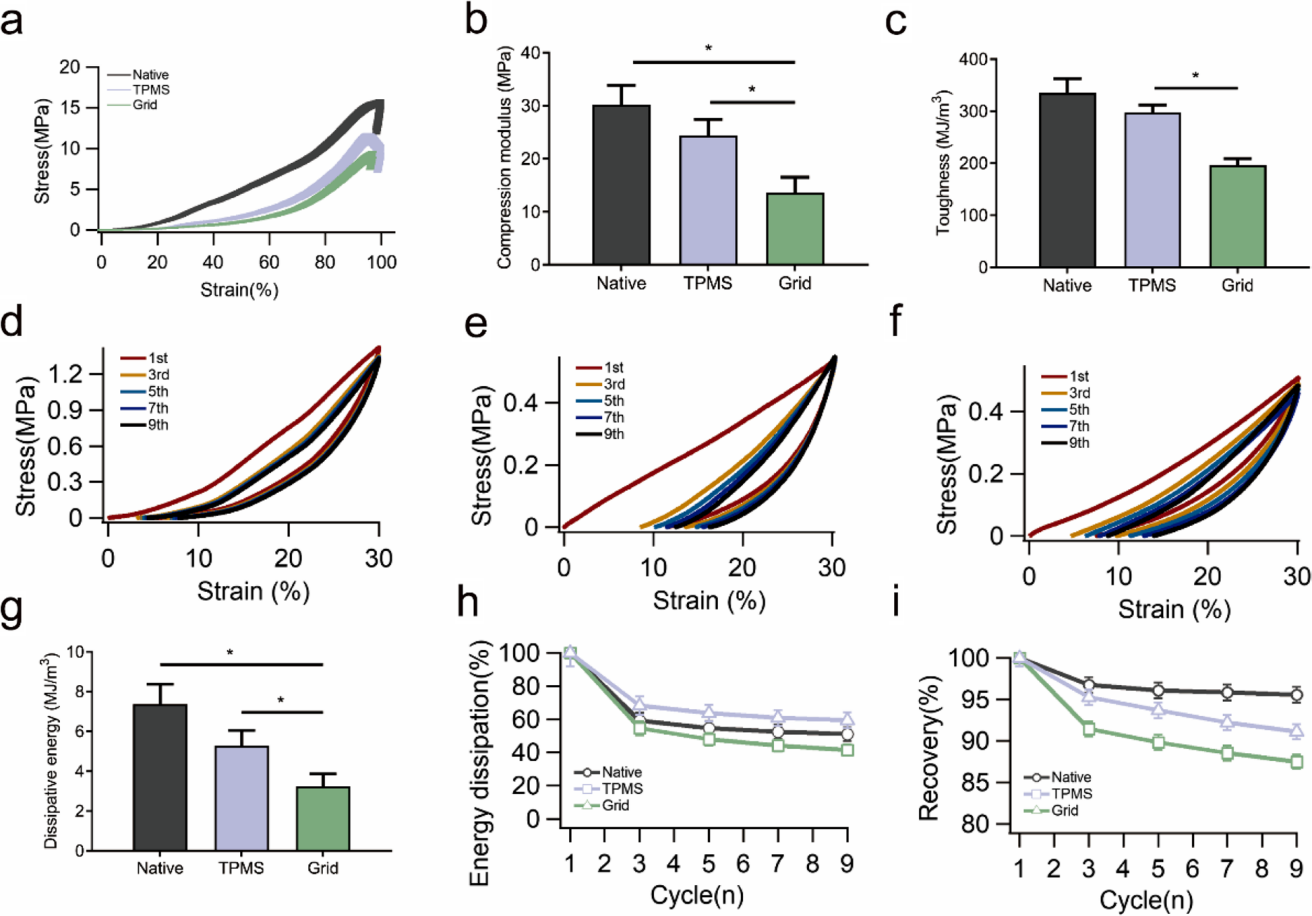
The results of the compression tests in the outer region, *n* = 3. **a** The stress–strain curve of the compression test. **b** The compression modulus in the three scaffolds, *P < 0.05. **c** The toughness in the three scaffolds, *P < 0.05. **d** Five compression-relaxation cycles in the native meniscus. **e** Five compression-relaxation cycles in the TPMS group. **f** Five compression-relaxation cycles in the grid group. **g** The dissipative energy of the 1st cycle in the three groups, *P < 0.05. **h** The percentage of dissipated energy in nine continuous compression-relaxation cycles. **i** The recovery percentage in nine continuous compression-relaxation cycles

The compression results of outer region are illustrated in Fig. 7, which are similar to those in the inner region, and the native meniscus had the highest limit of compressive stress at more than 15 MPa (Fig. 7a), as well as the largest compression modulus (Fig. 7b) and toughness (Fig. 7c). Among the above-mentioned results, a significant difference was found between the TPMS and grid groups. The hysteresis and energy dissipation were obvious in the three groups (Figs. 7d-f). The separated cycles are presented in Figures S9d-f, in which the first cycle in the TPMS group showed a distinctly different curve compared with other curves, indicating the large energy dissipation in this cycle. Although the TPMS group demonstrated a strong energy dissipation capacity based on the stress–strain curve, the native meniscus still had the largest dissipative energy in the first cycle (Fig. 7g). In addition, the TPMS group remained the largest percentage of dissipative energy after 10 cycles (Fig. 7h), which was 8% and 18% higher than native meniscus and grid group, respectively. The native meniscus kept more than 95% of the recovery capability, and the grid scaffold presented an obvious decrease to about 87% (Fig. 7i). The numerical values of these results are listed in Table 4.

**Table 4:** The compressive mechanical behavior of the regenerated meniscus in scaffolds

		Limit compression stress (MPa)	Compression modulus (MPa)	Toughness (kJ/m^3^)	Energy dissipation (kJ/m^3^)
Inner region	Native	9.75	19.23 ± 3.32	163.26 ± 12.26	9.34 ± 0.95
	TPMS	7.24	13.45 ± 2.96	131.49 ± 8.89	3.97 ± 0.54
	Grid	6.63	12.77 ± 2.72	122.96 ± 9.63	2.53 ± 0.41
Outer region	Native	15.63	30.20 ± 3.67	335.27 ± 27.55	7.32 ± 1.01
	TPMS	11.23	24.41 ± 3.05	297.59 ± 14.69	5.29 ± 0.77
	Grid	9.22	13.61 ± 2.92	196.39 ± 12.38	3.24 ± 0.63

**Fig. 8:**
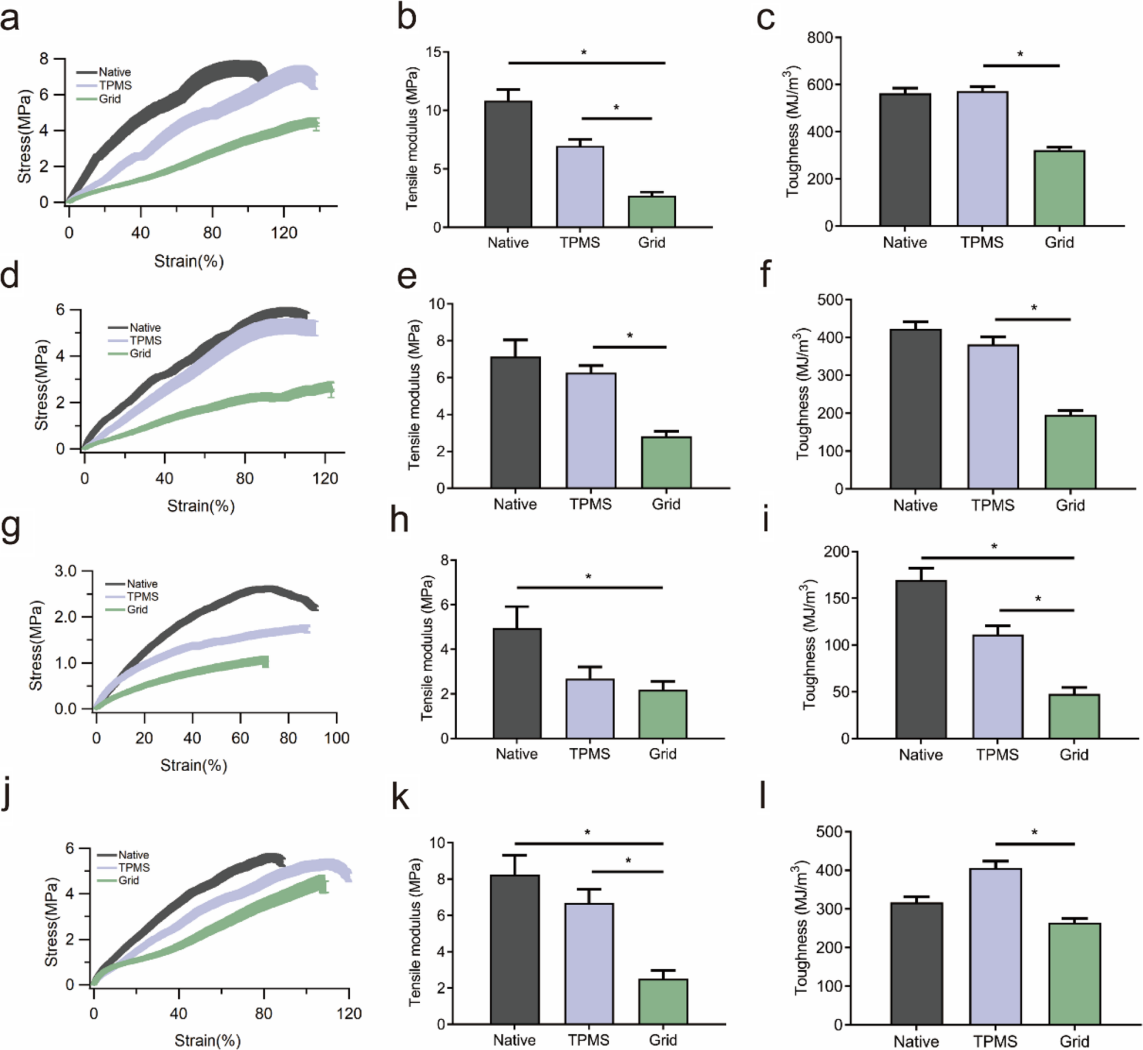
The results of the tensile tests in the three groups, *n* = 3. **a** The stress–strain curve of the tensile test in the bulk region. **b** The tensile modulus in the three groups in the bulk region, *P < 0.05. **c** The toughness in the three groups in the bulk region, *P < 0.05. **d** The stress–strain curve of the tensile test at the radial direction. **e** The tensile modulus in the three groups at the radial direction, *P < 0.05. **f** The toughness in the three groups at the radial direction, *P < 0.05. **g** The stress–strain curve of the tensile test in the inner region. **h** The tensile modulus in the three groups in the inner region, *P < 0.05. **i** The toughness in the three groups in the inner region, *P < 0.05. **j** The stress–strain curve of the tensile test in the outer region. **k** The tensile modulus in the three groups in the outer region, *P < 0.05. **l** The toughness in the three groups in the outer region, *P < 0.05

The results of tensile tests are exhibited in Fig. 8, in which the native meniscus showed the best performance in all the tests, and the TPMS-based scaffold demonstrated an appropriate mechanical behavior that was similar to the native meniscus.

In detail, the value of limit stress in the bulk tensile test in native meniscus and the TPMS group was close (about 7 MPa), while the grid group had the largest limit strain of more than 130% (Fig. 8a). A significant difference in the bulk tensile modulus could be found between each pair of groups (10.83 vs. 6.96 vs. 2.71 MPa) (Fig. 8b). Besides, the statistical difference in diversity of toughness was only existed between the TPMS and grid groups, and no discrepancy could be observed between the native meniscus and TPMS group (Fig. 8c). A similar phenomenon has been presented in the limit radial direction tensile stress and strain (Fig. 8d). In contrast to the bulk tensile test, the TPMS group and native meniscus had no notable diversity in tensile modulus (7.14 vs. 6.26 MPa, Fig. 8e) and toughness (Fig. 8f), while both groups had a significant difference to the grid group. For the inner region, the native meniscus had the largest limit stress of 2.62 MPa, and the TPMS group had the largest strain of more than 87% (Fig. 8g). Therefore, the native meniscus obtained the largest modulus of 4.95 MPa, and no significant difference was existed between the TPMS and grid groups (Fig. 8h). However, a notable discrepancy in toughness could be found between each pair of groups (Fig. 8i). The tendency of limit stress and strain for outer region was as same as that in the inner region, and the native meniscus and TPMS group had the largest numerical values of 5.59 MPa and 110.75%, respectively (Fig. 8j). A statistical diversity of modulus has been noted between each pair of groups (Fig. 8k), and the TPMS group owned the largest toughness (Fig. 8l). The detailed numerical values of the above-mentioned results are shown in Table 5.

**Table 5:** The tensile mechanical behavior of the regenerated meniscus in scaffolds

		Limit tensile stress (MPa)	Limit stain (%)	Tensile modulus (MPa)	Toughness (kJ/m^3^)
Bulk	Native	7.46	98.77	10.83 ± 0.95	563.94 ± 21.98
	TPMS	7.18	128.18	6.96 ± 0.57	572.51 ± 19.73
	Grid	4.47	137.73	2.71 ± 0.31	322.56 ± 12.21
Radial direction	Native	5.92	99.07	7.14 ± 0.90	423.11 ± 18.79
	TPMS	5.29	101.58	6.26 ± 0.39	382.04 ± 19.67
	Grid	2.71	122.89	2.82 ± 0.27	195.50 ± 12.17
Inner region	Native	2.62	72.24	4.95 ± 0.97	169.52 ± 12.77
	TPMS	1.75	87.60	2.68 ± 0.53	111.23 ± 9.39
	Grid	1.08	70.27	2.18 ± 0.38	47.81 ± 6.73
Outer region	Native	5.59	82.97	8.24 ± 1.08	316.76 ± 14.68
	TPMS	5.33	110.75	6.69 ± 0.75	406.05 ± 17.33
	Grid	4.54	107.12	2.51 ± 0.47	264.76 ± 10.69

## Discussion

To overcome the therapeutic obstacle of the meniscus injury and to improve the long-term clinical efficacy, fabrication of an appropriate meniscal scaffold is a promising solution. The present study assessed the application of the TPMS method in designing the porous meniscal scaffold. In order to further verify the feasibility of this scaffold, we assessed the status of load transmission in knee joint using the FEA, and the swine model was utilized to evaluate the real effects of meniscal replacement. This was a systematic research that integrated the material engineering and clinical medicine. As the function of meniscus is closely associated with stress and load, fabricating an engineered scaffold with remarkable mechanical properties may be beneficial for promoting meniscal regeneration and protecting articular cartilage. Therefore, a design method with the capability of regulating mechanical properties by adjusting pore structure parameters is essential. The mathematical model-based porous structure design method, especially the TPMS method, could achieve the above-mentioned objectives. Although several studies have explored the application of porous scaffolds in meniscal regeneration, few of them have introduced a mathematical modeling method to ameliorate the mechanical properties of scaffolds. In our previous studies, we have employed the FEA to prove that the compression and shear stress applied to the cartilage were related to the porous features [20], and the stress concentration could be effectively reduced through adjusting the modeling parameters of TPMS [32]. In the present study, we further confirmed the practicability of using this porous meniscal scaffold for meniscal replacement.

After 3 months of implantation, all the porous scaffolds were filled with newly born tissue, and the shape of the tissue-wrapped scaffold was meniscus, which indicated that the meniscal regeneration was achievable by the scaffold. In addition, the regenerated meniscus by the TPMS-based scaffold also demonstrated admirable mechanical properties, especially in the tensile tests. The histological staining explained this phenomenon, in which the outer and intermedia region mainly contained collagen I and a small amount of GAG in the TPMS-based scaffold. In the grid group, the expression of collagen II was higher than the native and TPMS groups, indicated that the content mismatch of grid group and healthy meniscus. Besides, the existence of collagen I was more evident in the TPMS-based scaffold as the stress distribution on it was similar to native meniscus. This type of collagen fibers was also the main component in the outer region of the native meniscus [33]. It can assist meniscus to resist against circumferential tension and extrusion during suffering load [34]. The lack of collagen I may cause the function loss in resisting tensile load in the outer and intermedia region of meniscus. As the dominant load in the normal mechanical environment of meniscus, tensile load had momentous effects on the regeneration of meniscus tissue [33]. It could upregulate the mRNA expression of collagen I and inhibit the expressions of pro-inflammatory factors induced by interleukin-1β (IL-1β) and tumor necrosis factor-α (TNF-α), thereby enhancing the production of ECM [35, 36]. Therefore, the realization of tensile mechanical properties similar to natural meniscus is very important for the successful reconstruction of porous meniscal scaffolds. Moreover, the native meniscus-like ECM in the TPMS group also led to the similar compression modulus and toughness to native meniscus. In contrast to the outer region, both TPMS-based and grid scaffolds exhibited a difference from the native meniscus in the inner region, and the expression of GAG, a component that can help the meniscus to resist against compression, was absent in this region [33]. The discrepancy in the inner compression modulus between porous scaffolds and native meniscus was presumed to be caused by this situation.

Although the mismatch in compression modulus was existed in the TPMS-based scaffold, it still plays a critical role in protecting cartilage due to its appropriate tensile modulus. According to the FEA results, the stress distribution on the bony structures and cartilage was significantly different from the healthy knee when a solid or grid meniscal scaffold was implanted. The abnormal stress distribution in the knee joint was caused by the meniscal dysfunction, which indicated that such structures cannot achieve the normal stress transmission and load buffering. The TPMS-based scaffold could reverse the negative phenomenon, in which the stress distribution and concentration area were similar to the healthy knee, and even the numerical values of the peak stress also exhibited the same variation tendency. The biomimetic stress and load characteristics may be beneficial for maintaining the integrity of articular cartilage. The subsequent in vivo study directly proved the FEA results. Due to the overload on cartilage, clear pathological changes were existed in the grid group. Meanwhile, cartilage in the TPMS group showed a normal histomorphology. The cause for this phenomenon could be attributed to the relationship of compressive load and tissue metabolism. For the cartilage tissue, the overload may lead to the aggravate of catabolic activity. Briefly, the catabolic pathway increased the activity of NF-κB, and the overexpression of cyclooxygenase-2 (COX2) and inducible nitric oxide synthase (iNOS) [37] resulted in the loss of GAG and disorder of collagen network [38]. The consequent chondrocyte apoptosis and hypertrophic may stimulate the process of osteoarthritis [39, 40].

According to the above-mentioned findings, the TPMS-based porous scaffold could either promote the meniscus-like tissue regeneration or improve the load transmission and protect the cartilage. The basement of these functions was the remarkable mechanical properties of the TPMS-based porous scaffold. Compared with the solid or grid scaffold, the stress applied to the TPMS-based scaffold could be effectively reduced and uniformly transferred to the bottom. The reason for this phenomenon could be related to the higher mechanical strength and relative density induced by the larger volume fraction and longer functional periodicity [32]. In addition, the volume fraction and functional periodicity exhibited to have influences on the energy dissipation. The higher energy dissipation capability resulted in a better load buffering ability of the scaffold. Normally, the energy dissipation is related to the porosity, while the pore shape is more significant, especially the Poisson’s ratio [41]. In the present study, the results indicated that the special deformed TPMS unit obtained a stronger energy dissipation capability than square pore, and even the grid scaffold had a higher porosity. Meanwhile, pore size at the stressed end and pore distribution also affected the energy dissipation [42], however, few studies have systemically explored the relationship among these parameters. For the deformed P structure, the larger pore size with a lower porosity was resulted in a higher energy dissipation. According to the FEA results of the static compression test, we supposed that the diversity of energy dissipation could be determined by the stress conduction behavior affected by the pore size and pore density.

Several limitations of the present study should be pointed out. Firstly, as mentioned above, the tensile stress plays a critical role in the fibrocartilage regeneration, such as anti-inflammation and fibrogenesis. Secondly, the relationship among the pore structure, stress, and tissue regeneration was not figured out. The research on the cell signaling pathways can make a better understanding about how the TPMS structure can affect the meniscal regeneration. Thirdly, the relationship between the porous structure and its mechanical properties was not deeply explored. A systemic study about the influences of pore characteristic parameters on mechanical behaviors will be advantageous to guide the design of meniscus or other scaffolds using tissue engineering. Fourthly, we did not employ any bioactive factors to cooperate with the scaffold. Due to the discrepancy in the medial and lateral regions of meniscus, loading different bioactive factors in the inner and outer regions of the scaffold may promote the anisotropic reconstruction of the meniscus.

## Conclusions

In the present study, we designed the meniscal scaffold using the TPMS method. A deformed P structure was employed as the basic unit, and scaffolds with different volume fractions and functional periodicities were fabricated by this unit. We analyzed the load buffering effect and mechanical properties through the FEA, and proposed an in vivo model to compare the effects of the TPMS-based scaffold and the traditional grid scaffold on cartilage protection and meniscal regeneration. The results were admirable, the TPMS-based scaffold demonstrated meniscus-like mechanical properties, the articular cartilage exhibited a better performance than the grid group. In addition, the regenerated meniscus tissue in the TPMS group obtained mechanical properties similar to those in the native meniscus, which indicated that the meniscal function was improved in the TPMS-based scaffold. The proposed method is highly promising for designing meniscal scaffolds using tissue engineering.

## Supplementary Information

**Additional file 1.**

## Data Availability

The datasets used and/or analysed during the current study are available from the corresponding author on reasonable request.
