# Supplementary material for: The triply periodic minimal surface-based 3D printed engineering scaffold for meniscus function reconstruction
Source: Biomater Res. 2022 Sep 17;26:45. doi: 10.1186/s40824-022-00293-3 (PMC9482755; doi:10.1186/s40824-022-00293-3)
Supplement: Supplementary file 1 — Additional file 1. [file 40824_2022_293_MOESM1_ESM.docx]

**The triply periodic minimal surface-based 3D printed engineering scaffold for meniscus function reconstruction**

Lan Li^a,b^†, Peng Wang ^a,b^†, Jing Jin^a^, Chunmei Xie^c^, Bin Xue^d^, Jiancheng Lai^e^, Liya Zhu^f^*, Qing Jiang^a,b^*

^a^ State Key Laboratory of Pharmaceutical Biotechnology, Division of Sports Medicine and Adult Reconstructive Surgery, Department of Orthopedic Surgery, Branch of National Clinical Research Center for Orthopedics, Drum Tower Hospital affiliated to Medical School of Nanjing University, Nanjing, 210000, China.

^b^ Jiangsu Engineering Research Center for 3D Bioprinting, Nanjing, 210000, China.

^c^ Hangzhou Lancet Robotics Company Ltd, Hangzhou, 310000, China.

^d^ National Laboratory of Solid State Microstructures, Department of Physics, Nanjing University, Nanjing, 210093, China.

^e^ Department of Chemical Engineering, Stanford University, Stanford, CA94305-6104, USA

^f^ School of Electrical and Automation Engineering, Nanjing Normal University, Nanjing, 210023, China.

† Lan Li and Peng Wang contributed equally to this work.

*Correspondence to:

Qing Jiang, MD, PhD, State Key Laboratory of Pharmaceutical Biotechnology, Division of Sports Medicine and Adult Reconstructive Surgery, Department of Orthopedic Surgery, Drum Tower Hospital affiliated to Medical School of Nanjing University, Nanjing University, No.321 Zhongshan Road, Nanjing, 210000, China. E-mail: qingj@nju.edu.cn

Liya Zhu, PhD, School of Electrical and Automation Engineering, Nanjing Normal University, No.1 Wenyuan Road, Nanjing, 210000, China. E-mail: 61193@njnu.edu.cn


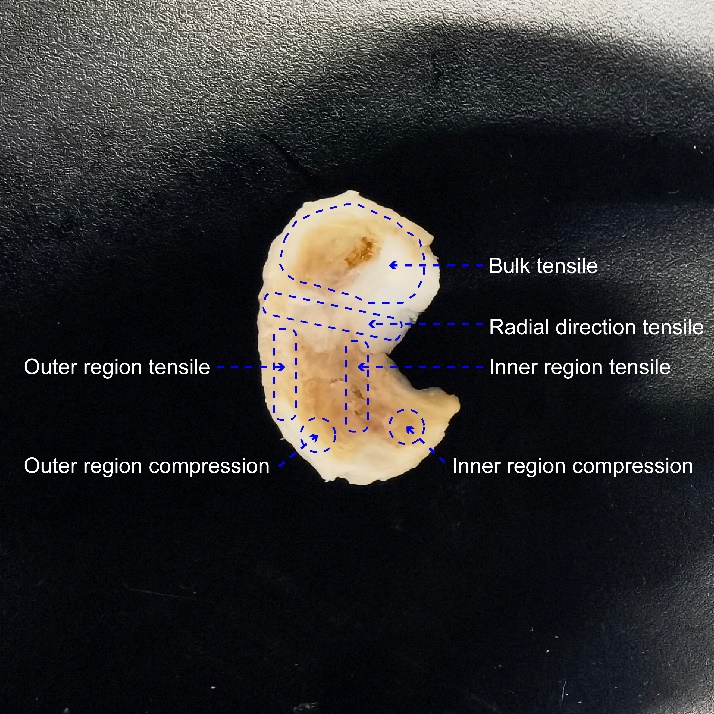


**Figure S1**. The diagram of mechanical test for the regenerated meniscus.


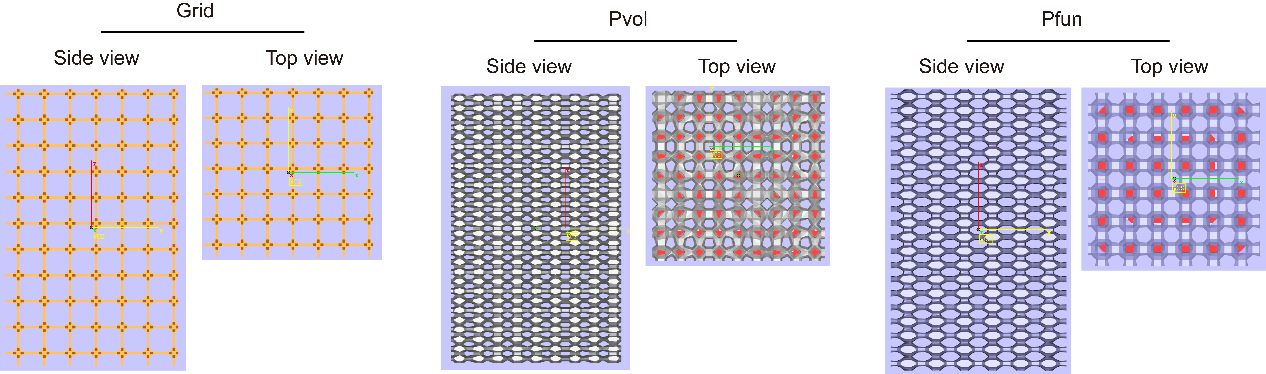


**Figure S2**. The diagram of mechanical test for the different porous scaffolds.


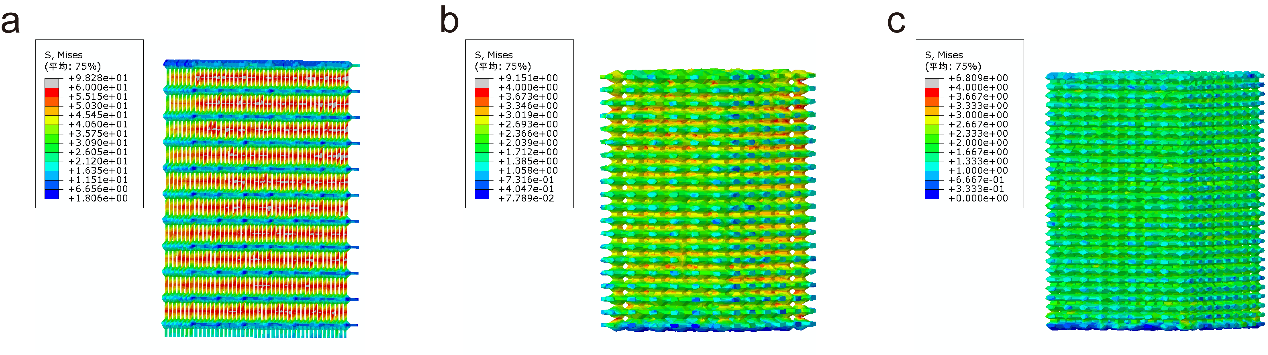


**Figure S3**. The FEA results of the different porous scaffolds under static compression. (a) Grid scaffold, (b) Pvol scaffold, (c)Pfun scaffold.


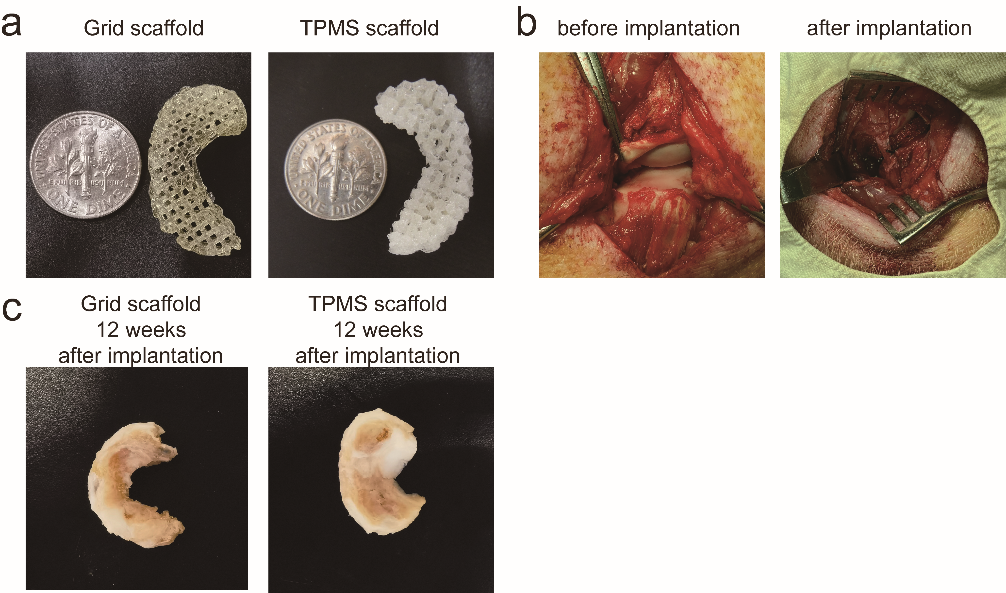


**Figure S4**. The general view of the meniscus and surgical process. (a) Porous scaffold for implantation, (b) The surgical process of the meniscus replacement, (c) The regenerated meniscus on the scaffolds.


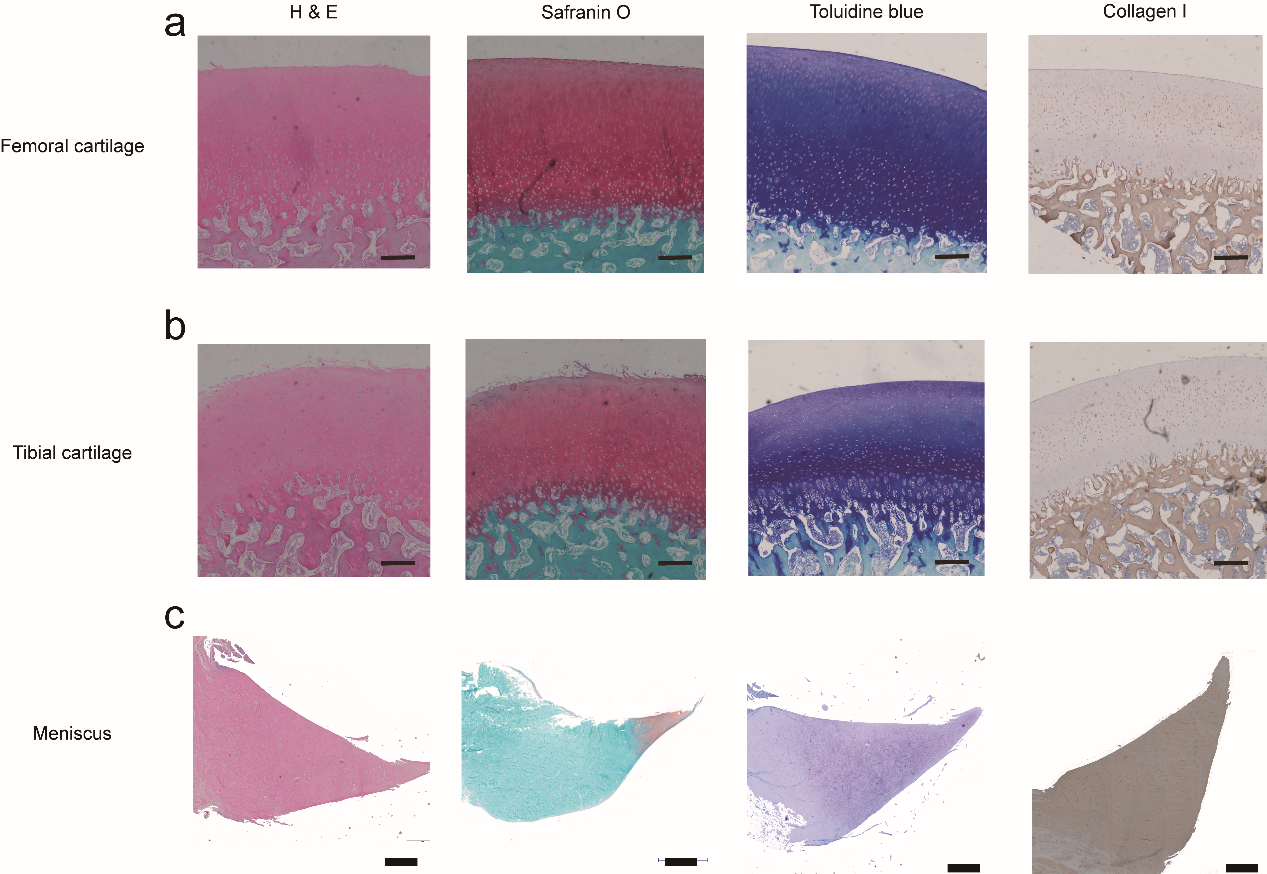


**Figure S5**. The histological staining of healthy tissues. (a) Femoral cartilage, scale bar: 500 μm, (b) Tibial cartilage, scale bar: 500 μm, (c) Meniscus, scale bar: 1000 μm..


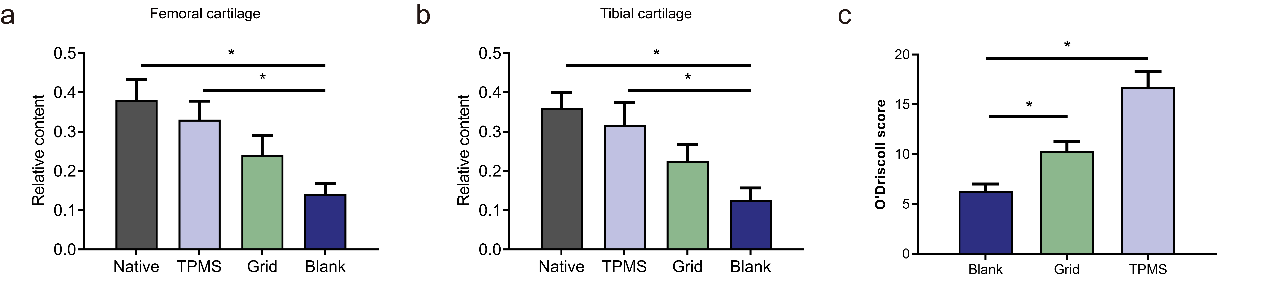


**Figure S6**. The quantitative analysis results of the cartilage. (a) The semi quantitative analysis of the GAG content for femoral cartilage, (b) (a) The semi quantitative analysis of the GAG content for tibial cartilage, (c) O’Driscoll score of the cartilage tissues.


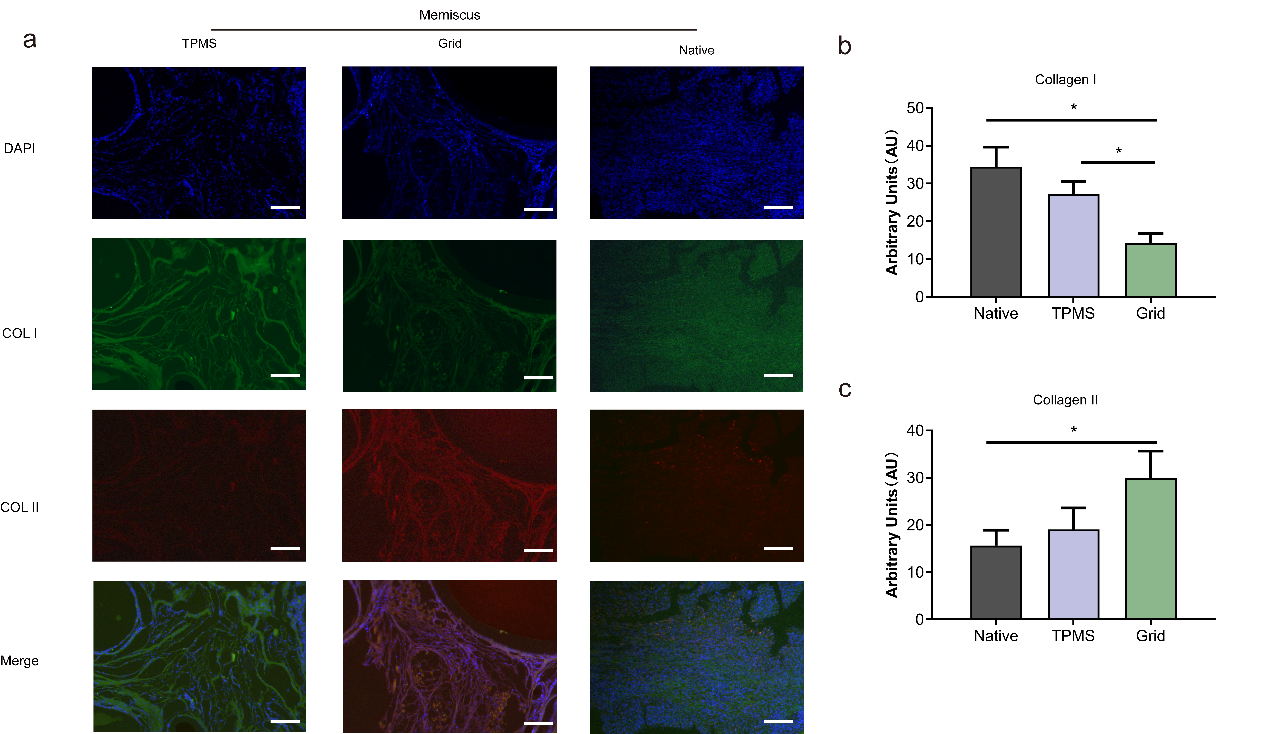


**Figure S7**. The results of immunofluorescence. (a) The immunofluorescences of DAPI, collagen I, collagen II, and the merge images for TPMS, grid, and native meniscus, scale bar: 500 μm,(b) The semi quantitative analysis of green fluorescence intensity, (c) The semi quantitative analysis of red fluorescence intensity.


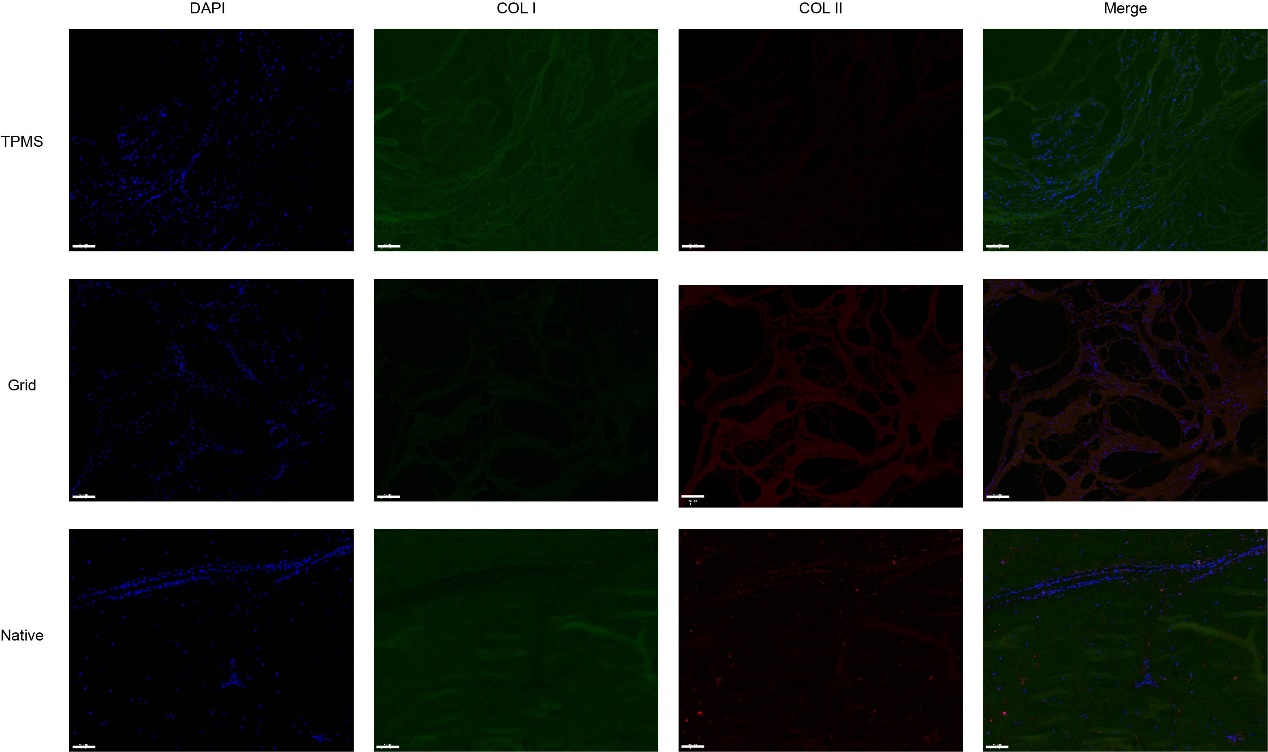


**Figure S8**. The results of immunofluorescence. scale bar: 50 μm.


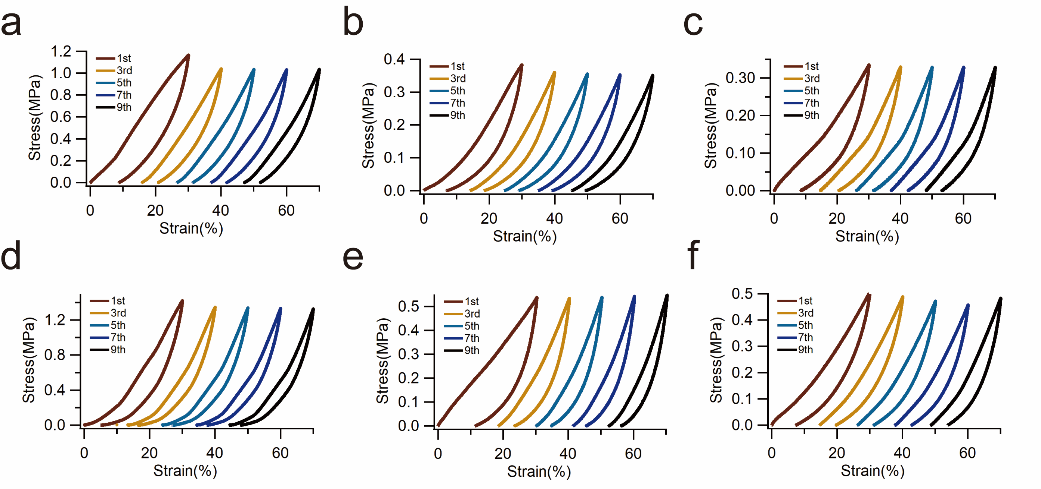


**Figure S9**. The separation stress-strain curves of the multi-cycle compression test for the inner and outer region. (a) Inner region of the native meniscus, (b) Inner region of the TPMS group, (c) Inner region of the grid group, (d) Outer region of the native meniscus, (e) Outer region of the TPMS group, (c) Outer region of the grid group.

**Table S1**. Material constants of the ligaments (ACL: Anterior cruciate ligament; PCL: Posterior cruciate ligament; LCL: Lateral collateral ligament; MCL: Medial collateral ligament; PT: Patellar tendon).

|  | C_1_ (MPa) | | C_3_ (MPa) | C_4_ (-) | C_5_ (MPa) | D_1_ (MPa^-1^) | λ* (-) |
| --- | --- | --- | --- | --- | --- | --- | --- |
| ACL | | 1.95 | 0.0139 | 116.22 | 535.039 | 0.00683 | 1.046 |
| PCL | | 3.25 | 0.1196 | 87.178 | 431.063 | 0.0041 | 1.035 |
| LCL | | 1.44 | 0.57 | 48.0 | 467.1 | 0.00126 | 1.063 |
| MCL | | 1.44 | 0.57 | 48.0 | 467.1 | 0.00126 | 1.063 |
| PT | | 3.25 | 0.1196 | 87.178 | 431.063 | 0.0041 | 1.035 |

**Table S2**. ICRS scoring system

| Cartilage repair evaluation | Points |
| --- | --- |
| Degree of defect repair |  |
| In level with surrounding cartilage | 4 |
| 75% repair of defect depth | 3 |
| 50% repair of defect depth | 2 |
| 25% repair of defect depth | 1 |
| 0% repair of defect depth | 0 |
| Integration to border zone |  |
| Complete integration with surrounding cartilage | 4 |
| Demarcating border <1 mm | 3 |
| 3/4th of graft integrated, 1/4^th^ with a notable border > 1 mm width | 2 |
| 1/2 of graft integrated with surrounding cartilage, 1/2 with a notable border >1 mm | 1 |
| From no contact to 1/4th of graft integrated with surrounding cartilage | 0 |
| Macroscopic appearance |  |
| Intact smooth surface | 4 |
| Fibrillated surface | 3 |
| Small, scattered fissures or cracks | 2 |
| Several, small or few but large fissures | 1 |
| Total degeneration of grafted area | 0 |
| Overall repair assessment |  |
| Grade I: normal | 12 |
| Grade II: nearly normal | 11-8 |
| Grade III: abnormal | 7-4 |
| Grade IV: severely abnormal | 3-1 |

**Table S3**. O’Driscoll scoring system

| Histological evaluation | Points |
| --- | --- |
| Nature of the predominant tissue |  |
| Cellular morphology |  |
| Hyaline articular cartilage | 4 |
| Incompletely differentiated mesenchyme | 2 |
| Fibrous tissue or bone | 0 |
| Safranin O staining of the matrix |  |
| Normal or nearly normal | 3 |
| Moderate | 2 |
| Slight | 1 |
| None | 0 |
| Structural characteristics |  |
| Surface regularity |  |
| Smooth and intact | 3 |
| Superficial horizontal lamination | 2 |
| Fissures – 25 to 100 per cent of thickness | 1 |
| Severe disruption, including fibrillation | 0 |
| Structural integrity |  |
| Normal | 2 |
| Slight disruption, including cysts | 1 |
| Severe disintegration | 0 |
| Thickness |  |
| 100 % of normal adjacent cartilage | 2 |
| 50-100% of normal cartilage | 1 |
| 0-50% of normal cartilage | 0 |
| Bonding to adjacent cartilage |  |
| Bonded at both ends of graft | 2 |
| Bonded at one end, or partially at both ends | 1 |
| Not bonded | 0 |
| Freedom from cellular changes of degeneration |  |
| Hypocellularity |  |
| Normal cellularity | 3 |
| Slight hypocelluarity | 2 |
| Moderate hypocelluarity | 1 |
| Severe hypocelluarity | 0 |
| Chondrocyte clustering |  |
| No clusters | 2 |
| <25% of the cells | 1 |
| 25-100% of the cells | 0 |
| Freedom from degenerative changes in adjacent cartilage |  |
| Normal cellularity, no clusters, no staining | 3 |
| Normal cellularity, mild clusters, moderate staining | 2 |
| Mild or moderate hypocellularity, slight staining | 1 |
| Severe hypocellularity, poor or no staining | 0 |

**Table S4**. Peak stress (MPa) applied in the knee joint

|  | Femur | Femoral cartilage | Tibia | Tibial cartilage | Meniscus |
| --- | --- | --- | --- | --- | --- |
| Pfun | 13.80 | 2.09 | 15.53 | 2.19 | 7.53 |
| Pvol | 13.72 | 2.02 | 12.90 | 2.01 | 3.84 |
| Grid | 28.37 | 4.42 | 41.86 | 4.58 | 3.49 |
| Native | 11.48 | 2.09 | 15.43 | 1.96 | 6.98 |
| Solid | 23.39 | 3.70 | 34.39 | 3.99 | 0.85 |
